# The Medical Society of the State of New York

**Published:** 1893-03

**Authors:** 


BUFFALO MEDICAL AND SURGICAL JOURNAL

A MONTHLY REVIEW OF MEDICINE AND SURGERY.

*EDITORS:*

THOMAS LOTHROP, M. D. - - WM. WARREN POTTER, M. D.

All communications, whether of a literary or business character, should be addressed to the managing editor: 284 FRANKLIN STREET, BUFFALO, N. Y.

VOL. XXXII. MARCH, 1893. No. 8.

### THE MEDICAL SOCIETY OF THE STATE OF NEW YORK.

THE eighty-seventh annual meeting of this society, held at Albany, February 7, 8, and 9, 1893, under the presidency of Dr. Lewis S. Pilcher, of Brooklyn, will rank as one of the most interesting and remarkable meetings in its history. In the first place, considerable attention was attracted to this meeting, all over the country, on account of the action of the American Medical Association, at Detroit, at its meeting held in June, 1892. It will be remembered that a committee of five was appointed to treat with a like committee of the Medical Society of the State of New York, on the subject of a renewal of reciprocal relations between these two medical bodies.

Most unwisely some foolhardy enthusiast moved to invite the New York State Medical Association to appoint a committee to act with these two on the subject, which was carried. But how, by any process of ratiocination whatsoever, it could come about that the New York State Medical Association could have anything to do with the matter, is beyond the comprehension of the most acute observer. It was absolutely and morally certain when a committee from the New York State Medical Association was added that the Medical Society of the State of New York would decline to have anything to do with the affair in any shape or manner, and this was probably the reason why the association was added as a party to the proposed treaty.

The meeting just held has shown that the predictions of friends of both societies, that is, the Medical Society of the State of New York and the American Medical Association, were correct when they said that the Medical Society of the State of New York would not condescend to appoint a committee to treat with the other two associations on the subject.

The Medical Society of the State of New York, at its recent meeting, further emphasized its opinion on the questions that have been the subject of controversy for the past ten years, by striking out from its by-laws all references to codes whatsoever; so now, instead of being a so-called new-code society, it is absolutely and unalterably a "no-code" society. This seems to us the most fitting answer that could be returned to the American Medical Association, for its outrageous treatment of the physicians from the State of New York who have seen fit for one reason and another to attend its meetings during the past ten years.

The fact is that the American Medical Association has been governed by a ring, for the past decade, that has known nothing except ethics gone mad in its management. It is to be hoped that the newer generation will take hold of affairs and rehabilitate the fossilized management of Messrs. Davis, Toner, and McGuire. The latter gentleman, who is now President of the American Medical Association, advocated in his annual address to the American Surgical Association, a few years ago, an abrogation of codes. To be consistent, he must do likewise in his address to the American Medical Association, in Milwaukee, next June. We shall see.

But we digress. It is to President Pilcher that the Medical Society of the State of New York tenders its most heartfelt thanks for the successful manner in which he conducted this whole subject, and for the able presentation of it in his inaugural address. It was so faithfully and thoroughly set forth in his masterful address as to leave in the minds of the society only one idea, namely, the propriety of wiping out all codes, and in future to stand on the broad foundation of gentlemanly conduct the one towards the other.

Thus the Medical Society of the State of New York at last comes to occupy the ground that was so ably advocated by Dr. D. B. St. John Roosa in 1882, when he proposed a substitute for the then pending new-code proposition, in the nature of a resolution, which, if adopted, would have at once made a no-code society of that body.

The scientific work of the society at this meeting was fully up to its highest standard, and reflects great credit on the management from the president down.

In selecting Dr. Herman Bendell, of Albany, for the succession, the society has honored one of its most faithful and devoted members, who has always worked hard for its best interests. We predict that President Bendell will preside over a meeting in 1894 not second to any that has heretofore been held.

